# Complete Genome Sequence from an Uncultivated Freshwater *Elusimicrobiota* Lineage

**DOI:** 10.1128/mra.00426-22

**Published:** 2022-08-11

**Authors:** Franziska Cremer, Lucas Serra Moncadas, Adrian-Stefan Andrei

**Affiliations:** a Faculty of Science, University of Zurich, Zurich, Switzerland; b Microbial Evogenomics Lab, Limnological Station, Department of Plant and Microbial Biology, University of Zurich, Kilchberg, Switzerland; Montana State University

## Abstract

Here, we report the first complete genome of an uncultivated freshwater *Elusimicrobiota* organism recovered from a nonaxenic *Amoebozoa* sp. culture. The chromosome was obtained from a metagenomic long-read sequencing run and was assembled as a circular element at a 47× coverage, a length of 3.8 Mbp, and a G+C content of 68.6%.

## ANNOUNCEMENT

*Elusimicrobiota* encompass an enigmatic branch of the prokaryotic tree of life that was designated following the isolation of the obligately anaerobic beetle gut-associated Elusimicrobium minutum (1). Despite the organisms’ recent characterization in diverse animal-associated and natural environments (2), inferences on their lifestyle strategies and metabolic capacities are hindered by lack of high-quality genomic data.

An *Amoebozoa* sp. was enriched from the perialpine Lake Zurich (406 m above sea level [a.s.l.], 47°18′N, 8°34′E, Switzerland). The organism was maintained in tissue culture flasks filled with 50 mL of autoclaved Swiss Aplina (Pearlwater, Termen, Switzerland) water and fed with cyanobacterium Planktothrix rubescens strain A7. Three nonaxenic stationary-phase cultures were filtered through 5- and 0.2-μm-pore-size polycarbonate filters to concentrate the microbial biomass. The 0.2-μm filters were cut into small pieces and subjected to DNA extraction using the MagAttract HMW DNA kit (Qiagen, Hilden, Germany). The obtained DNA was further purified with Beckman Coulter AMPure XP magnetic beads and subsequently used for metagenomic sequencing on a Nanopore PromethION platform using a FLO-PRO002 (R9.4.1) flow cell. The sequencing one-dimensional (1D) library was constructed with the SQK-LSK110 ligation sequencing kit (ONT, Oxford, UK) in conformity with manufacturer’s instructions without any prior DNA fragmentation or size selection.

Obtained raw reads (approximately 2 million reads; mean read length of 9 kbp, median read quality of 11.7, *N*_50_ of 14.4 kbp) were basecalled and quality trimmed (Q score 7) with Guppy 5.1.15 prior to assembly with Flye 2.9-b1768 (3). The recovered circular chromosome (3,802,950 bp; 47× coverage) was classified using the GTDB-Tk v1.4.0 toolkit (4) and by comparing its 16S rRNA gene (predicted with barrnap 0.9) with the SILVA database (version 138) (5). Potential genome contamination was assessed by CheckM v1.1.3 (6). Coding DNA sequences and tRNAs were predicted by Prokka 1.12 (7) and NCBI’s PGAP pipeline. BlastKOALA (8) was used to assign KO identifiers to orthologous genes. Inferences of complete metabolic pathways and general biological functions were conducted with the online KEGG mapping tools (https://www.genome.jp/kegg/kegg1b.html) using summarized KO numbers. PFAM domains were identified using the script pfam_scan.pl with the PFAM database release 32 (9). The growth rate was assessed, by exploiting codon usage patterns, with the R package gRodon (10), while antiphage systems were predicted with DefenseFinder (11). Default parameters were used except where otherwise specified.

The genome (strain JAD_PAG50586_1) was classified as belonging to an uncultivated order within *Elusimicrobiota* by both GTDB (UBA1565) and SILVA (Lineage IV) databases. It was found to contain 4,148 coding sequences (CDS) and 53 tRNAs and to possess 1 rRNA operon. The genome-inferred metabolic reconstructions depicted a diderm bacterium with a low growth rate (as assessed by gRodon) and an aerobic heterotrophic lifestyle. Small neutral and branch-chain amino acid transporters can alleviate auxotrophies (i.e., impaired biosynthesis for 7 proteinogenic amino acids) and can contribute (through degradation pathways; l-leucine) together with glycolysis (Embden-Meyerhof pathway) to replenishing the acetyl coenzyme A (acetyl-CoA) pool needed to fuel the tricarboxylic acid (TCA) cycle.

### Data availability.

All sequence data are available through the National Center for Biotechnology Information (NCBI) via the BioProject accession numbers PRJNA824509 (CP096137.1, GCA_023213235.1, and SRX14779367). Additional proteome annotations (KEGG, Prokka, and Pfam) are available in the figshare repository: https://doi.org/10.6084/m9.figshare.20358804.
